# Priming with DNA Expressing Trimeric HIV V1V2 Alters the Immune Hierarchy Favoring the Development of V2-Specific Antibodies in Rhesus Macaques

**DOI:** 10.1128/JVI.01193-20

**Published:** 2020-12-22

**Authors:** Santhi Devasundaram, Margherita Rosati, Antonio Valentin, Svenja Weiss, Vincenza Itri, Hung V. Trinh, Jenifer Bear, Bhabadeb Chowdhury, Celia C. LaBranche, David Montefiori, Guido Ferrari, Mangala Rao, Xiang-Peng Kong, Susan Zolla-Pazner, George N. Pavlakis, Barbara K. Felber

**Affiliations:** a Human Retrovirus Pathogenesis Section, Vaccine Branch, Center for Cancer Research, National Cancer Institute at Frederick, Frederick, Maryland, USA; b Human Retrovirus Section, Vaccine Branch, Center for Cancer Research, National Cancer Institute at Frederick, Frederick, Maryland, USA; c Department of Medicine, Division of Infectious Diseases, Icahn School of Medicine at Mount Sinai, New York, New York, USA; d U.S. Military HIV Research Program, Walter Reed Army Institute of Research, Silver Spring, Maryland, USA; e Henry M. Jackson Foundation for the Advancement of Military Medicine, Inc., Bethesda, Maryland, USA; f Department of Surgery, Duke University, Durham, North Carolina, USA; g Duke Human Vaccine Institute, Duke University, Durham, North Carolina, USA; h Department of Molecular Genetics and Microbiology, Duke University, Durham, North Carolina, USA; i Department of Biochemistry and Molecular Pharmacology, NYU School of Medicine, New York, New York, USA; Ulm University Medical Center

**Keywords:** HIV, DNA vaccine, Env, V1V2, cyclic V2, gp145, antibody, linear peptide, rhesus macaque, prime-boost, ADCP, ADCC, C1q, NAb

## Abstract

The RV144 vaccine trial revealed a correlation between reduced risk of HIV infection and the level of nonneutralizing-antibody (Ab) responses targeting specific epitopes in the second variable domain (V2) of the HIV gp120 envelope (Env) protein, suggesting this region as a target for vaccine development. To favor induction of V2-specific Abs, we developed a vaccine regimen that included priming with DNA expressing an HIV V1V2 trimeric scaffold immunogen followed by booster immunizations with a combination of DNA and protein in rhesus macaques. Priming vaccination with DNA expressing the HIV recombinant subtype CRF01_AE V1V2 scaffold induced higher and broader V2-specific Ab responses than vaccination with DNA expressing CRF01_AE gp145 Env. Abs recognizing the V2 peptide that was reported as a critical target in RV144 developed only after the priming immunization with V1V2 DNA. The V2-specific Abs showed several nonneutralizing Fc-mediated functions, including ADCP and C1q binding. Importantly, robust V2-specific Abs were maintained upon boosting with gp145 DNA and gp120 protein coimmunization. In conclusion, priming with DNA expressing the trimeric V1V2 scaffold alters the hierarchy of humoral immune responses to V2 region epitopes, providing a method for more efficient induction and maintenance of V2-specific Env Abs associated with reduced risk of HIV infection.

**IMPORTANCE** The aim of this work was to design and test a vaccine regimen focusing the immune response on targets associated with infection prevention. We demonstrated that priming with a DNA vaccine expressing only the HIV Env V1V2 region induces Ab responses targeting the critical region in V2 associated with protection. This work shows that V1V2 scaffold DNA priming immunization provides a method to focus immune responses to the desired target region, in the absence of immune interference by other epitopes. This induced immune responses with improved recognition of epitopes important for protective immunity, namely, V2-specific humoral immune responses inversely correlating with HIV risk of infection in the RV144 trial.

## Abstract (precis)

The aim of this work was to design and test a vaccine regimen focusing the immune response on targets associated with infection prevention. We demonstrated that priming with a DNA vaccine expressing only the HIV Env V1V2 region induces Ab responses targeting the critical region in V2 associated with protection. This work shows that V1V2 scaffold DNA priming immunization provides a method to focus immune responses to the desired target region, in the absence of immune interference by other epitopes. This induced immune responses with improved recognition of epitopes important for protective immunity, namely, V2-specific humoral immune responses inversely correlating with HIV risk of infection in the RV144 trial.

## INTRODUCTION

The human immunodeficiency virus (HIV) RV144 vaccine clinical trial, using a canarypox vector (ALVAC) expressing HIV genes (encoding Gag/protease and a membrane-bound gp120 Env) as a priming immunization and ALVAC plus recombinant HIV gp120 Env glycoproteins (AIDSVAX B/E) as a booster immunization, showed a modest (31.2%) vaccine efficacy (1). Analysis of correlates of risk of infection identified nonneutralizing antibodies (Abs) targeting the Env variable V1V2 region and Abs able to mediate cellular cytotoxicity as vaccine-induced immune responses significantly linked to protection (2–6). The V1V2 region is located at the apex of the Env glycoprotein trimer (reviewed in references 7 and 8) and can form a five-stranded beta-barrel structure (9–13) comprising A, B, C, C′, and D strands. The presence of V2 Abs responses targeting a specific epitope (amino acids [aa] 170 to 176; HXB2 numbering) that represents the C strand region within the beta-barrel (10) was confirmed by different approaches, including sieve analysis (3) and analysis of binding to linear peptides, cyclic V2, and gp70-V1V2 scaffolds (5, 6, 14, 15). Several macaque vaccine challenge studies support the role of V2-specific Ab in reducing the risk of simian immunodeficiency virus (SIV) (16–21) or simian-human immunodeficiency virus (SHIV) (22, 23) acquisition. It was also found that different vaccine platforms induced only low levels of V2-specific Ab responses in macaques vaccinated with different HIV Env proteins (24).

To mimic the V1V2 conformation within the native Env trimer, immunogens using V1V2 protein sequences engrafted onto trimeric scaffold proteins (25) or glycopeptide scaffolds from the V1/V2 domain expressed with mannose-5 glycans (26, 27) were developed. A vaccine combining the V1V2 trimeric scaffold protein and DNA expressing the complete gp120 induced robust cross-clade V1V2-specific Abs in rabbits and rhesus macaques (28, 29). This macaque study established that vaccines including V1V2 scaffold proteins were able to induce humoral responses and focus them on epitopes recognized by Abs associated with protection in the RV144 trial.

In another line of research, we established strategies to focus cellular immune responses on subdominant regions of HIV/SIV proteins. Vaccine regimens using plasmid DNA expressing only conserved elements (CE) of HIV as a priming immunization induced immune responses targeting subdominant epitopes of Gag (30–32) or Env (33), which are otherwise inefficiently recognized in the presence of immunodominant epitopes produced by the full-length immunogen. In the present study, we extended this vaccine concept to focus the development of humoral immune responses on structurally conserved regions of HIV Env.

To focus Ab responses on the HIV-1 V2 region, we used a vaccination regimen that included priming with DNA encoding a trimeric V1V2 scaffold protein followed by DNA-protein coimmunization boosts. The use of a nucleic acid-based vaccine is a simple method allowing efficient expression of a structurally defined immunogen which results in the development of both humoral and cellular immunity (reviewed in references 34–36) that can be maintained for long periods and can be boosted by the same or heterologous boosting strategies (37). This protocol recapitulates our previous experiences in HIV vaccine development, as follows. (i) First, the immune responses are focused on subdominant regions of the HIV proteome which alters the immune response hierarchy by priming with DNA expressing highly conserved regions (CE) of Gag or Env; these responses are greatly augmented by booster vaccination with DNA expressing CE and full-length immunogens (30–33). (ii) Next, DNA-protein is coadministered in the same anatomical sites to induce cellular and humoral immune responses of high magnitude and longevity that efficiently disseminate to mucosal sites (21, 38–42), a strategy corroborated by others (28, 43–45). Macaques vaccinated with the DNA-protein coimmunization regimen showed a delay in SIV/SHIV acquisition and efficient control of viremia, preventing progression toward AIDS (21, 38, 46).

In this report, we show that priming with DNA encoding a trimeric HIV V1V2 immunogen is more efficient than vaccination with DNA expressing the complete Env molecule in focusing the Ab responses on the V2 epitopes associated with reduced risk of infection, as reported in the RV144 trial. These data indicate that selected immunogens and vaccine strategies can be used to alter immune hierarchy and favor responses to regions associated with protective immunity.

## RESULTS

### Selection and characterization of the V1V2 scaffold DNA immunogen.

**FIG 1:**
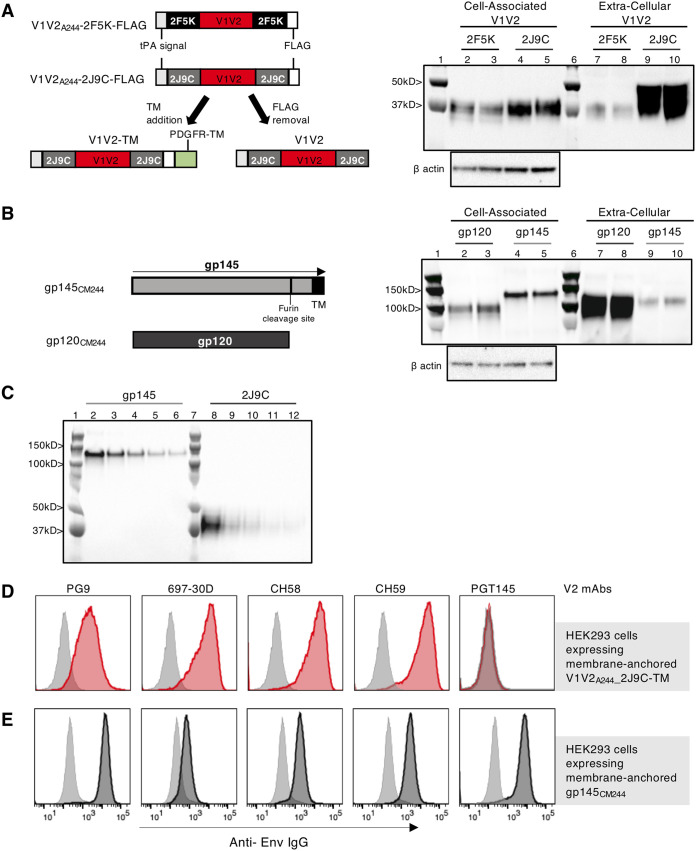
Development of DNA vaccine to target immune responses to V2 of HIV Env. (A) Design and expression of V1V2 scaffolds. The cartoons show the details of two different protein scaffolds (2F5K and 2J9C) containing the 80-aa region of V1V2_A244_, the N-terminal tPA signal peptide, and the C-terminal FLAG-tag (left). The cartoon also depicts the FLAG-tagged V1V2-2J9C protein with the PDGFR transmembrane domain (V1V2-TM) used for *in vitro* studies (plasmid 447H) and the V1V2-2J9C DNA used for the vaccine studies, with the FLAG tag removed (plasmid 418H). HEK293T cells were transfected with V1V2_A244_-2J9C-FLAG DNA and V1V2_A244_-2F5K-FLAG DNA, and 2 days later, cells and supernatants were analyzed by Western blotting by loading 1/200 of each fraction, and the membranes were probed with anti-FLAG–HRP Ab. Lanes 1 and 6, Kaleidoscope prestained protein marker; lanes 2, 3, 7, and 8, V1V2_A244_-2F5K; lanes 4, 5, 9, and 10, V1V2_A244_-2J9C. Two independent DNA plasmid clones from each construct were tested (lanes 2 and 3, lanes 4 and 5, lanes 7 and 8, and lanes 9 and 10). Equal loading of the cell-associated fraction was controlled by probing the membrane with a β-actin Ab. (B) CM244 Env was expressed as cell-associated gp145 and as a soluble monomeric gp120 protein from the CMV promoter. The cartoon indicates the furin cleavage site and the TM region in gp145. HEK293T cells were transfected with two independent clones of each DNA. Two days later, cells and supernatants were analyzed by Western blotting by loading 1/200 of the fractions. The membrane was probed with a mixture of plasma from macaques vaccinated with HIV Env followed by anti-monkey IgG–HRP. The ECL detection method was used to visualize the expressed proteins. Lanes 1 and 6, Kaleidoscope prestained protein marker; lanes 2, 3, 7, and 8, gp120_CM244_; lanes 4, 5, 9, and 10, gp145_CM244_. (C) Expression levels of the endotoxin-free plasmid preparations used for macaque vaccination producing gp145 (plasmid 416H) or V1V2-2J9C (plasmid 418H) after 24 h upon transfection in HEK293T cells. Twofold serial dilutions (1:2 to 1:16) of total proteins were analyzed, and the membrane was probed with the V2-specific CH58 MAb. Lanes 1 and 7, Kaleidoscope prestained protein marker; 2 to 6, gp145; lanes 8 to 12, V1V2-2J9C. (D and E) Stable HEK293H cell lines were generated expressing V1V2_A244_-2J9C anchored to the cell membrane via PDGFR-TM (D) and membrane-anchored trimeric gp145_CM244_ (E). Histogram overlays show the binding of selected V2-specific MAbs (PG9, 697-30D, CH58, CH59, and PGT145) to V1V2_A244_ (red histograms) (D) and membrane-anchored trimeric gp145 (black histograms) (E). Gray-shaded histograms show the binding of the anti-p24^Gag^ MAb 241-D, used as a negative control. The results show recognition by (i) PG9 (V2q-type MAb), supporting the quaternary structure of the trimeric V1V2 in a glycan-dependent fashion; (ii) 697-30D (V2i-type MAb), recognizing a discontinuous epitope overlapping the α_4_β_7_ integrin binding motif (LDI/V); and (iii) CH58 and CH59 (V2p-type MAbs), isolated from RV144 trial volunteers known to target linear and cyclic peptides spanning aa 170 to 176 of V2.

To select an optimal immunogen able to focus the humoral responses on V1V2, the HIV-1 clade AE strain A244 V1V2 coding sequence was grafted into two previously described trimeric protein scaffolds (2F5K and 2J9C) (25). The plasmids encoding these two molecules, which also included a FLAG tag, were analyzed for expression upon transient transfection (Fig. 1A). Comparison of the FLAG-tagged V1V2_A244_-2F5K and -2J9C scaffolds showed that the V1V2_A244_-2J9C protein was better expressed and accumulated primarily in the extracellular compartment, whereas the V1V2_A244_-2F5K protein remained mainly cell associated. Therefore, vaccine studies were performed using the V1V2_A244_-2J9C variant without the FLAG tag, referred to hereinafter as V1V2_A244_. Transfection of gp145_CM244_ DNA showed expression of cell-associated gp145 and the furin-cleaved extracellular gp120 (Fig. 1B).

Protein production from the gp145_CM244_ and V1V2_A244_-2J9C plasmids used in the vaccine study was compared in the same transfection experiment. Total protein production was analyzed by Western immunoblotting probing the membrane with the V2p-specific monoclonal antibody (MAb) CH58. Analysis of serially diluted samples showed similar levels of protein expression from these plasmid DNAs (Fig. 1C).

To verify the conformation of the V1V2_A244_ region within this scaffold, a stable HEK293 cell line was generated expressing V1V2-TM protein (Fig. 1A) comprising a fusion of V1V2_A244_-scaffold to the transmembrane domain (TM) of the platelet-derived growth factor receptor (PDGFR). A stable cell line expressing the membrane-anchored gp145_CM244_ (Fig. 1B) was used for comparison. A panel of conformation-specific V2 MAbs, including PG9, 697-30D, CH58, and CH59, was used to assess the exposure and conformation of the V1V2 domain on the cell surface by binding experiments and flow cytometry. The binding of MAbs to membrane-anchored V1V2-TM (Fig. 1D) was compared to that of gp145 (Fig. 1E). Recognition by PG9 (V2q-specific MAb), and 697-30D (V2i-specific MAb) supported a trimeric V2 structure having a beta-strand configuration of the C strand (47) (Fig. 1D). Recognition by CH58 and CH59 Ab (V2p-specific Ab isolated from RV144 trial volunteers) indicated that the C strand is also present in its alpha-helical configuration. These data are consistent with the structural polymorphism of the alpha-helical and beta-strand V1V2 configurations of the C strand region of V1V2 (10), which is reflected here by the binding of the V2q and V2i MAbs. On the other hand, PGT145, which requires a quaternary structure dependent on the presence of constant region C1 and variable region V3 (48), failed to bind to the V1V2_A244_-TM, as expected (Fig. 1D), while it recognized the gp145_CM244_ protein expressed on the surface of the HEK293 control cell line (Fig. 1E). Together, these data show that the V1V2 protein expressed from the DNA vector has the proper trimeric folding and flexible conformations with beta-strand and alpha-helical structures.

### DNA vaccine regimen.

**FIG 2:**
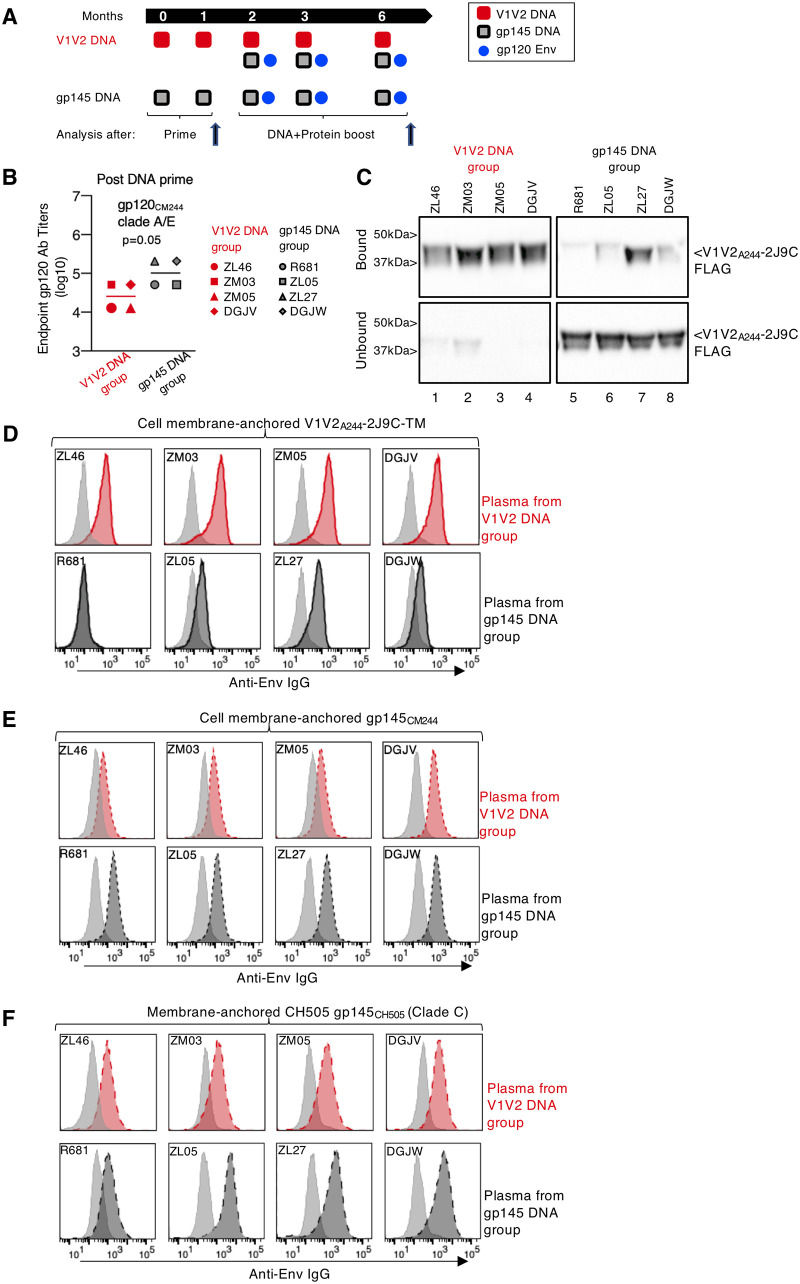
Induction of V1V2-specific Abs by priming vaccination with DNA expressing secreted V1V2_A244_ scaffold. (A) Schematic representation of the vaccination regimens, including two DNA primes (months 0 and 1) and three DNA-protein coimmunization boosts (months 2, 3, and 6) in two groups of macaques (*n* = 4). As the prime, the V1V2 DNA group received V1V2_A244_-2J9C DNA and the gp145 DNA group received gp145_CM244_ DNA. For boosts, both groups received gp145_CM244_ DNA, and the V1V2 DNA group continued to receive V1V2_A244_-2J9C DNA. The animals also received gp120_CM244_ protein adjuvanted with GLA-SE as part of the boost. Ab responses were analyzed after the prime and after the boost, as indicated. (B) Endpoint titers of the vaccine-induced plasma Ab to gp120_CM244_ after the prime, as measured by ELISA. The *P* value is from a parametric *t* test. (C) Immunoprecipitation, performed under nondenaturing conditions with HEK293-produced FLAG-tagged V1V2_A244_-2J9C protein and macaque plasma samples collected after administration of two doses of each of the DNA primes. The antigen-Ab complexes that had bound to protein A-Sepharose beads were analyzed on 12% denaturing gels. Protein A-Sepharose-bound (50% of sample) and unbound (25% of sample) materials were loaded onto gels, and the membranes were probed with anti-FLAG–HRP Ab. Bands labeled “bound” reveal the presence of V1V2-2J9C-FLAG in antigen-Ab complexes, whereas the bands labeled “unbound” show the absence of Abs in plasma specific for V1V2-2J9C-FLAG. Lanes 1 to 4, V1V2 DNA group; lanes 5 to 8, gp145 DNA group. (D to F) Histogram overlays show flow cytometry analysis of plasma Ab binding to membrane-anchored Env. Binding of plasma from the V1V2 DNA group (top) and the gp145 DNA group (bottom) to the stable HEK293H cell line expressing membrane-anchored V1V2-TM (D), gp145_CM244_ (E) and gp145_CH505_ (F) is shown. Data from individual animals from the V1V2 DNA (red histograms) and gp145 DNA (dark gray histograms) vaccine groups are shown. Binding of prebleed plasma from the corresponding animals is also shown (light gray). Macaque Ab binding was measured using a PE-conjugated anti-human IgG MAb cross-reactive with macaque IgG.

Two groups of Indian rhesus macaques (4 per group) were used, each receiving two DNA-only priming vaccinations followed by three DNA-protein booster vaccinations (Fig. 2A). To focus the Ab responses on the V2 region of HIV-1 Env, one group of macaques was primed with a plasmid encoding V1V2_A244_-2J9C (referred to as the V1V2 DNA group), while the second group was primed with DNA encoding membrane-anchored gp145_CM244_ (referred to as the gp145 DNA group). The DNA vaccines were delivered via the intramuscular route followed by electroporation. As booster vaccinations (Fig. 2A), all animals were coimmunized with gp145_CM244_ DNA and the monomeric HEK293-produced gp120_CM244_ protein (Fig. 1B), adjuvanted with the Toll-like receptor 4 (TLR-4) agonist GLA-SE. The booster vaccine for the animals in the V1V2 DNA group included V1V2_A244_ DNA as well as the gp145_CM244_ DNA and the adjuvanted gp120_CM244_. Plasma samples were collected after the priming vaccination (2 weeks after the second vaccination; referred to here as “prime”) and after the third booster (2 weeks after the fifth vaccination; referred to here as “boost”).

### Focusing of immune responses on the V2 region by DNA vaccination.

The magnitude of vaccine-induced Ab responses after the prime in both groups was analyzed by enzyme-linked immunosorbent assay (ELISA) using gp120_CM244_ as the detection antigen. The V1V2 DNA prime induced Abs that recognize gp120 Env but to a lesser extent than Abs induced by the gp145 DNA prime (Fig. 2B), which is expected, since the V1V2 immunogen contains fewer Env epitopes than gp145. To evaluate whether the vaccine-induced Abs were able to recognize the trimeric V1V2 protein under physiological nondenaturing conditions, plasma samples from the immunized macaques were analyzed by immunoprecipitation. Samples from the V1V2 DNA vaccine group collected after the priming vaccinations efficiently recognized the V1V2_A244-2_J9C-FLAG protein (Fig. 2C, lanes 1 to 4). In contrast, plasma samples from the gp145 DNA-primed animals failed to significantly bind to the V1V2_A244_-2J9C-FLAG protein, which remained mainly in the unbound fraction (Fig. 2C, lanes 5 to 8). Only plasma from one of the four macaques from the gp145 DNA group, ZL27, showed some reactivity with the V1V2 scaffold protein but to a lower extent than the plasma from animals in the V1V2 DNA group. These data demonstrate a significant difference in the composition of the V1V2-specific Abs elicited by the two vaccine regimens and highlight that gp145 DNA priming induced poor V1V2-specific Abs compared to the V1V2 DNA prime.

We also examined the ability of these Abs to recognize the membrane-bound trimeric V1V2-TM and gp145 Env proteins on the surfaces of stable HEK293H cells (Fig. 2D and E, respectively). We found strong recognition of the V1V2-TM protein by plasma samples from the four macaques primed with the V1V2 DNA (Fig. 2D, top). In contrast, weaker binding to the V1V2-TM was observed using plasma from three of the four macaques primed with the gp145 DNA, while plasma from macaque R681 did not provide a positive signal at a 1:800 dilution in this flow cytometry assay (Fig. 2D, bottom). Overall, these data are in agreement with the immunoprecipitation data shown in Fig. 2C, with macaque ZL27 having the higher V1V2-specific Abs among the four animals from the gp145 DNA vaccine group.

The Abs elicited by the V1V2 DNA prime were also able to recognize membrane-bound trimeric gp145 Env from clade AE.CM244 (Fig. 2E, top) and the heterologous clade C.CH505 (Fig. 2F, top). This binding was slightly weaker than the binding observed with plasma from the gp145 DNA primed macaques (Fig. 2E and F, bottom), which is expected, since fewer epitopes are present in the V1V2 immunogen. Taken together, these data demonstrate that priming with the V1V2 DNA vaccine is more efficient than priming with the gp145 DNA vaccine in inducing Abs targeting both the soluble and membrane-bound forms of V1V2 in their trimeric conformation.

### Stronger recognition of V2 region by the V1V2 DNA vaccine-induced Abs is maintained after the DNA-protein boost.

**FIG 3:**
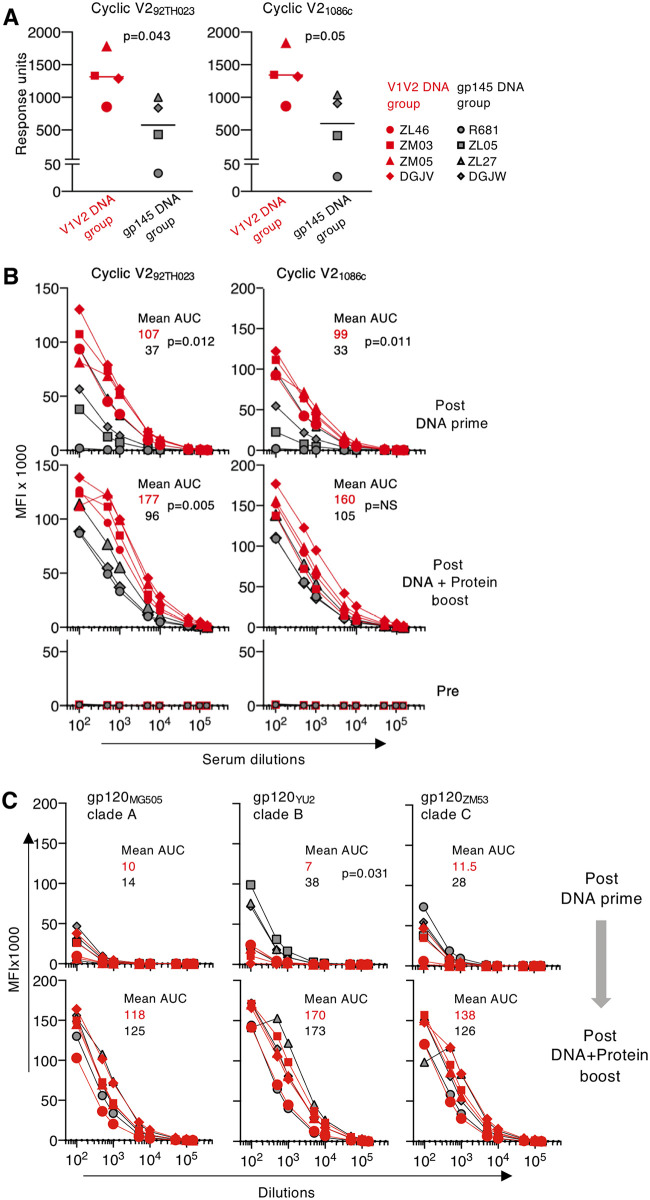
V1V2 DNA priming induced higher V2-specific Abs and gp120-specific Abs. (A) Plasma Ab binding (1:50 dilution) to cyclic V2 was measured by an SPR assay after the prime. Reactivity to cyclic V2 peptides from different clades (AE.92TH023 and C.1086) are reported as response units. Individual animals from the V1V2 (red symbols) and gp145 (black symbols) DNA-primed groups are shown. *P* values are from a parametric *t* test. (B and C) A Luminex assay shows Ab binding to cyclic V2 from different clades (AE.92TH023 and C.1086) (B) and to gp120 from different clades (A.MG505, B.YU2, and C.ZM53) (C) using serially diluted serum from the immunized macaques. Data are given as mean fluorescence intensity (MFI) for the V1V2 DNA group (red symbols) and the gp145 DNA group (black symbols) after the prime (top) and after the boost (middle [B] and bottom [C]). Values for prevaccination serum samples are shown (bottom [B]). The mean area-under-the-curve (AUC) values for the 2 vaccine groups (red and black lettering, respectively) after prime and after boost are shown. *P* values are from a parametric *t* test.

We next examined the ability of vaccine-induced Abs to recognize cyclic V2. We found efficient cross-clade recognition of the cyclic V2 peptides AE.92TH023 and C.1086 measured by surface plasmon resonance (SPR) (Biacore) (Fig. 3A) after the prime with significantly higher V2-specific Abs (*P* = 0.043 and *P* = 0.05, respectively) in plasma samples from the V1V2 DNA group than in plasma from the gp145DNA group. By a Luminex assay, using serially diluted sera collected after the prime, we again observed significantly higher binding to both clade AE and C cyclic V2 molecules in samples from the V1V2 DNA primed animals (Fig. 3B, top) than in samples from the gp145 DNA group (*P* = 0.012 and *P* = 0.011, respectively). The responses to cyclic V2 in these two assays were in agreement with the Abs’ ability to recognize V1V2 in the pulldown assay and to recognize membrane-bound V1V2 (Fig. 2C and D).

In both groups, the booster vaccinations combining DNA and protein resulted in greatly increased V2-specific Abs (Fig. 3B, middle) and of gp120-specific Abs (Fig. 3C, bottom), as measured by the Luminex assay. We found that animals from the V1V2 DNA prime group maintained significantly higher cyclic V2-specific responses to AE.92TH023 (*P* = 0.005) after the boost (Fig. 3B, middle).

### Identification of distinct Ab responses within the V2 region induced by the V1V2 DNA priming regimen.

**FIG 4:**
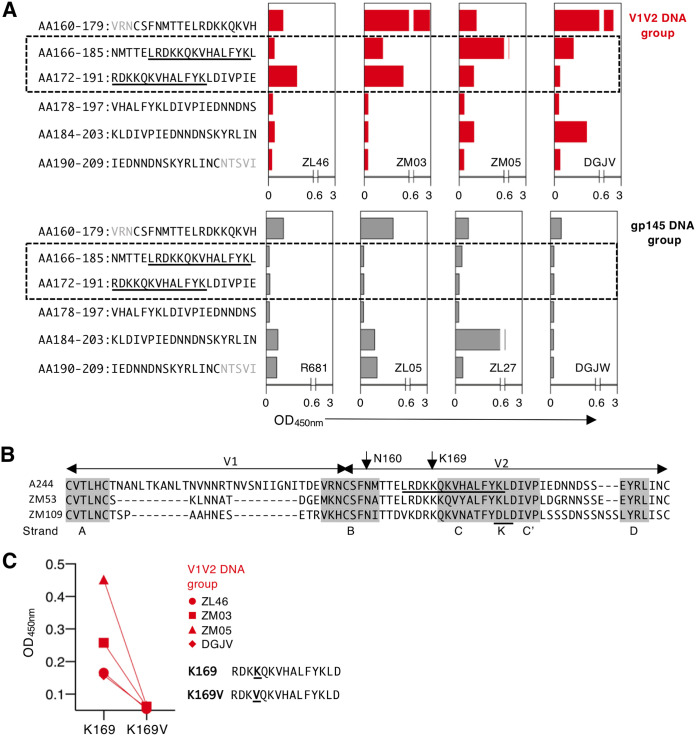
Distinct epitope recognition in V2 by Abs elicited by the two vaccine regimens. (A) ELISA analysis using linear peptides (20-mers overlapping by 14 aa) spanning the V2 region (amino acid numbering follows CM244 [GenBank accession number AAW57760]) was performed using a 1:50 plasma dilution after the DNA prime for each animal from both groups. (B) Alignment of V1V2 amino acid sequences of A244, ZM53, and ZM109. The five strands (A, B, C, C’, and D, and the helical turn K) composing the V1V2 beta-barrel are indicated by gray shading (10). Residue K169 (HXB2 numbering) was identified by sieve analysis in RV144 as mismatched in vaccine breakthrough infections (3). The peptide from aa 165 to 178 (LRDKKQKVHALFYK) is underlined and spans the residues shown to interact with Abs associated with reduced risk of infection from the RV144 trial (14). (C) Binding of plasma (1:50 dilution) from the immunized macaques to the wild-type V2 peptide and to its mutant (K169V) was measured by ELISA (HXB2 amino acid numbering). Individual plasma samples from V1V2 DNA animals were analyzed after the prime.

To map the vaccine-induced Ab responses within the V2 region, a linear peptide analysis was performed by ELISA using six overlapping peptides (20-mer peptides overlapping by 14 aa) covering the V2 region (Fig. 4A). The responses were measured for each animal from both vaccine groups. Upon priming, Abs induced by the V1V2 DNA showed strong reactivity with two peptides located at the N-terminal portion of V2 (aa 166 to 185 and 172 to 191) (Fig. 4A, top), while the gp145 DNA vaccine induced Ab responses with higher reactivity to two C-terminal peptides (aa 184 to 203 and 190 to 209) (Fig. 4A, bottom). Animals from both groups showed reactivity to the N-terminal peptide from aa 160 to 179. Interestingly, the reactivity to the peptides from aa 166 to 185 and 172 to 191, found with the V1V2 DNA group, represents recognition of a region which covers the C strand region of the V1V2 β-barrel structure (Fig. 4B) and comprises the sequence recognized by Abs associated with reduced risk of infection in the RV144 clinical trial (Fig. 4B, underlined amino acids). In contrast, plasma from the macaques primed with the gp145 DNA had minimal or no reactivity to these two peptides. This finding prompted us to explore whether this interaction was sensitive to the K169 mutation (Fig. 4B), identified by sieve analysis in breakthrough infections of RV144 (3). Comparison of binding to the wild-type peptide with K169 and mutant peptide K169V using plasma samples from the V1V2 primed macaques revealed complete abrogation of peptide recognition by the K169V mutation (Fig. 4C). These data demonstrate that the V1V2 DNA vaccine-induced Abs are able to recognize an important epitope associated with protective immunity in the RV144 trial. Together, the V1V2 DNA-primed Ab responses favor recognition of the B/C strands in the β-barrel structure, while gp145 DNA-induced responses preferentially recognize the D strand, showing a distinct hierarchy of responses within V2 peptides.

### Distinct peptide recognition within V2 is maintained upon booster vaccination.

**FIG 5:**
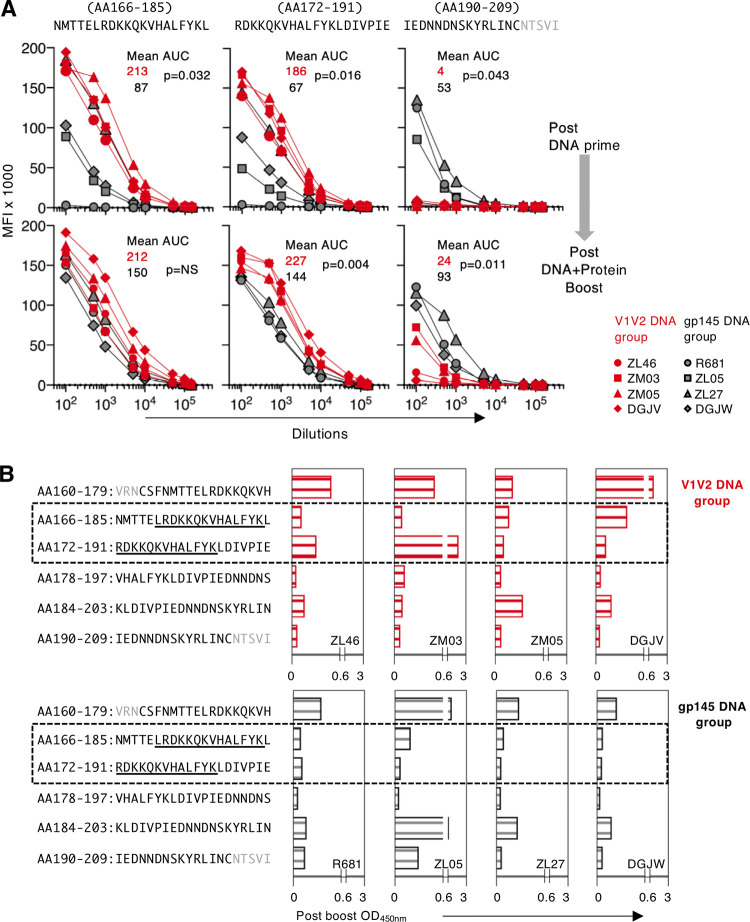
Distinct epitope recognition in V2 is maintained after the booster vaccination. (A) Ab binding to selected peptides was measured in a Luminex assay using serially diluted sera. Data obtained after the second prime (top) and after the last boost (bottom) from the V1V2 DNA group (red symbols) and gp145 DNA group (black symbols) are shown as MFI. The mean AUC values are given: red lettering indicates the V1V2 DNA group, and black lettering indicates the gp145 DNA group. *P* values are from a parametric *t* test. (B) ELISA analysis using linear peptides (20-mers overlapping by 14 aa) spanning the V2 region was performed as for Fig. 4A using a 1:50 plasma dilution of plasma collected after the DNA-protein booster vaccination from animals from both groups.

We further compared the vaccine-induced serum Ab binding to selected peptides spanning the V2 region using a Luminex assay. In samples collected after the prime, we found significantly higher Ab binding to the N-terminal peptides (aa 166 to 185 and 172 to 191) in animals in the V1V2 DNA group than in those in the gp145 DNA group (*P* = 0.032 and *P* = 0.016, respectively) (Fig. 5A, top). In contrast, higher binding to the C-terminal peptide (aa 190 to 209) was found in the sera from animals in the gp145 DNA group (*P* = 0.043) (Fig. 5A). Although responses to the N-terminal peptides (aa 166 to 191) in the gp145 DNA-primed animals increased upon the booster vaccinations, the recognition of both peptides, which contain the RV144 epitope, remained higher in the V1V2 DNA-primed macaques (*P* = 0.004 for peptide 172–191) (Fig. 5A, bottom).

In agreement with the Luminex data, mapping of the Ab binding to V2 peptides by ELISA showed stronger binding to the key peptides (aa 166 to 185 and 172 to 191) in samples from the animals in the V1V2 DNA group collected after the boost (Fig. 5B, top). Although the booster vaccination modestly increased the responses to these peptides in animals from the gp145 DNA-primed group, this increase did not reach the levels found in the animals from the V1V2 group (Fig. 5B, bottom). Similarly, higher responses to the C-terminal peptides (aa 184 to 203 and 190 to 209) were maintained in the animals from the gp145 DNA group. Together, comparison of the responses between the two vaccine groups showed that the differences in the hierarchy of recognized V2 peptides established by the prime vaccination were maintained after the boost.

### Distinct antibody effector functions induced by the vaccine regimens.

**FIG 6:**
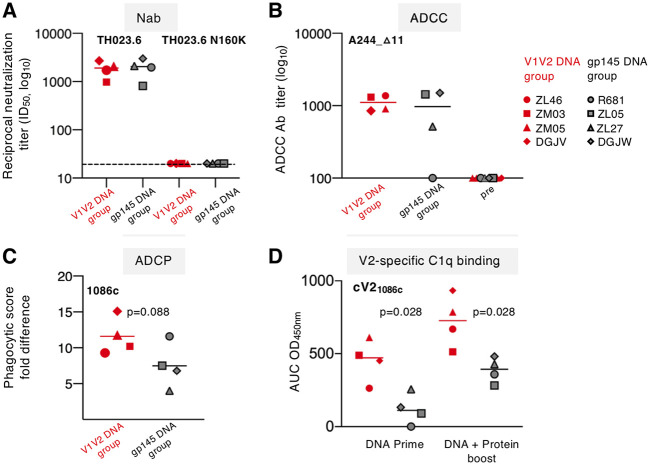
Distinct effector function of the Ab induced by the two vaccine regimens. (A) Neutralizing Abs (Nab) to tier 1A pseudovirus 92TH023.6 (left) and the mutant 92TH023.6N160K (right) were measured in serum from all the animals 2 weeks after the boost. Amino acid numbering is that for HXB2, and the location of N160 is indicated in Fig. 4B. The dashed line indicates the limit of detection of neutralization in the TZM-bl assay. (B) ADCC obtained with gp120_A244_-coated CEM-NKR target cells was measured in sera from V1V2-primed animals (red symbols) and gp145-primed animals (black symbols) after the last booster vaccinations. (C) Phagocytosis of gp140_1086c_-coated fluorescent beads by THP-1 cells triggered by antibodies induced after the prime in sera from V1V2 DNA group (red symbols) and the gp145 DNA group (black symbols). The fold differences were determined by dividing the ADCP score from immunized sera by the score from the respective prebled sera. *P* values are from a nonparametric *t* test. (D) C1q binding to V2-specific Abs was measured in the two vaccine groups after the prime and the boost, by ELISA using plates coated with cyclic V2_1086c_. *P* values are from a nonparametric *t* test.

The neutralizing activity of the vaccine-induced Abs was analyzed using the tier 1 92TH023.6 Env and its V2 N160K mutant. Sera collected from macaques from both vaccine groups after the booster vaccinations showed similar neutralization titers for 92TH023.6 in the TZM-bl assay (Fig. 6A). We found that this neutralizing activity was sensitive to the N160K mutation (Fig. 6A). This residue is located within the peptide from aa 160 to 179 in the N-terminal portion of V2 (Fig. 4B), which showed strong reactivity with plasma from both groups after the prime and boost (Fig. 4A and 5B). These data demonstrate that the V2-specific Abs recognizing this peptide are able to neutralize the tier 1-pseudotyped virus, and Abs with this feature were induced by both vaccine regimens.

Ab-dependent cellular cytotoxicity (ADCC) was measured by GranToxiLux (GTL) flow cytometry using gp120_A244_Δ11_-coated CEM.NKR_CCR5_ cells as targets. Although no ADCC activity was observed after DNA-only priming in either of the two vaccine regimens, ADCC was detected in all V1V2-primed animals and in three of four gp145 DNA-primed animals after the booster vaccinations (Fig. 6B). This finding is in agreement with our previous report that inclusion of an HIV/SIV Env protein component in the vaccine is necessary to induce the Ab levels required to mediate measurable ADCC activity (38, 41). Under these experimental conditions, using the complete Env as the target, we could not distinguish the contribution of the V2-specific Ab in ADCC.

We examined additional Fc-mediated effector functions, including antibody-dependent phagocytosis (ADCP) and C1q binding, two Ab features associated with nonneutralizing control of infection (reviewed in references 49 and 50) (Fig. 6C and D). ADCP was examined using sera collected after the DNA prime from both groups, allowing the functional characterization of the V2-specific Abs from the V1V2 DNA vaccine group. The uptake of fluorescent beads coated with gp140_1086c_ by THP-1 cells was measured by flow cytometry (Fig. 6C). These data demonstrate that Abs induced by the V1V2 DNA-primed macaques mediated higher levels of phagocytosis (median phagocytic score, 11.6) than Abs induced upon priming with the gp145 DNA vaccine (median phagocytic score, 7.4). Therefore, the V2-specific Abs induced upon priming with V1V2 DNA have improved phagocytic function.

We next examined the ability of C1q, the protein responsible for initiating the classical complement activation pathway, to bind to the Fc portion of the vaccine-induced V2-specific Abs using a two-step ELISA using cyclic-V2_1086c_-coated plates (Fig. 6D). C1q binding to the V2-specific Abs was significantly higher in the V1V2 DNA-primed animals than in the gp145 DNA-primed animals, and this difference was maintained after the boost, suggesting a stronger ability to activate the complement cascade by the Abs induced by the V1V2 DNA vaccine.

## DISCUSSION

The RV144 trial showed that nonneutralizing Abs targeting the V2 loop of HIV Env were associated with decreased risk of infection. Together with V1, the V2 region forms a complex five-stranded beta-barrel structure, and Ab recognition of a specific region within the V2 C strand was found to be critical for the observed protective immune response. The complex HIV Env structure renders many critical protein domains relatively inaccessible to Abs, and their exposure or occlusion depends on protein folding (reviewed in references 7 and 51–55) and transitions between different conformations; this applies to V2 as well as to many other regions in Env (56). As a result, exposed variable “decoy” Env epitopes and glycosylation can prevent the production and/or binding of Abs able to block infection. To reduce or prevent the development of responses to decoy epitopes, several vaccine approaches have been designed to redirect immune responses to distinct Env regions. One of those strategies is the development of constrained HIV Env molecules with native-like structures that display enhanced stability and favorable antigenic features. These constrained Env immunogens were achieved by several methods, including the use of (i) stabilized Env trimers (SOSIP) (57–60), (ii) gp120 with engineered outer domains (61), (iii) cleavage-independent native flexibly linked (NFL) Env trimers (62), and (iv) gp140 in a closed conformation displayed on ferritin nanoparticles (63). In addition, many alternative methods to focus the Ab response on protective epitopes have been used, including the use of distinct regions in Env that can be “isolated” from the remaining portions of Env while maintaining a native conformation; these include RC1, which facilitates the recognition of the glycan patch associated with the third variable region (V3) of gp120 (64); MPER peptides adjuvanted in liposomes, used in conjunction with a gp140 oligomer prime to induce Ab to the gp41 fusion intermediate (65); membrane-embedded formulations to induce MPER-targeting Abs (66, 67); and use of a cross-linked V1 and V2 (68), and trimerized gp120 (69), glycan-stabilized V1V2 scaffold (26, 27). Other approaches have been developed to specifically focus Ab responses on V2, including the design and use of (i) V1V2 scaffold protein immunogens together with gp120 DNA (25, 28, 29), (ii) a V2 loop peptide together with liposomal lipid A as an adjuvant (70), (iii) gp140 SOSIP or foldon trimers carrying signature-guided mutations of V2 broadly neutralizing monoclonal Abs (71), and (iv) a mutated V1V2 sequence which favors the β-strand conformation of the V2 C strand spliced onto the C terminus of murine leukemia virus gp70 (72).

In this study, we demonstrated in nonhuman primates that using a vaccine regimen consisting of an HIV DNA prime plus DNA-protein boost that includes priming with a plasmid expressing a soluble trimeric V1V2 scaffold protein is able to efficiently induce V2-specific Ab responses focused to a critical epitope within V2. Our data show that V1V2 DNA priming efficiently induces Abs that share features with the protective RV144 Abs and that recognize V2 in its trimeric configuration. In contrast, macaques primed with DNA expressing the complete HIV Env developed poor Ab responses targeting this epitope, indicating that responses to V2 are suboptimally induced when this region is presented within full-length Env. Thus, our data indicate that priming with V1V2 DNA alters the hierarchy of responses within V2 and that the strongly V2-specific Abs were maintained upon the DNA prime–DNA-protein boost. In fact, our results show that priming with DNA encoding gp145 elicits Ab responses preferentially targeting the C-terminal part of the V2 region—Abs that are not associated with protection. Moreover, the immunogenic epitope hierarchy that is established by the full-length Env protein is altered by priming with DNA encoding the V1V2 scaffold, thus providing a strategy that redirects the Ab responses toward the N-terminal portion of the V2 region and epitopes recognized by Abs that are associated with protection from HIV, SIV, and SHIV (47). Thus, priming with DNA expressing the trimeric V1V2 scaffold protein allows the immune system to focus the Ab responses to this target in the absence of any immune competition with other epitopes. This provides a critical advantage to the V1V2 vaccine regimen promoting the induction of desired Ab responses targeting epitopes documented to correlate with protection.

Immune interference among HIV epitopes has been observed in several studies. We found immune interference between Gag and Env epitopes and reported a potent negative impact of Env epitopes on the induction of Gag T cell responses in vaccinated macaques (73). Immunogenic competition between Gag/Pol and Env was also observed in a clinical trial (74). We identified highly conserved but subdominant regions within HIV and SIV Gag (31, 32) and Env (33) and demonstrated that priming with DNA vaccines expressing only these subdominant regions was able to alter the cellular immune hierarchy, resulting in potent recognition of these otherwise poorly immunogenic epitopes in rhesus macaques (30). Interestingly, for HIV Gag, we found that the regions containing the subdominant T cell epitopes also failed to elicit humoral responses and that prime-boost vaccination alleviated this impairment (30), a study that guided our concept that selective priming of the immune response is critical for eliciting humoral immune responses targeted to desired protein regions, as we showed in this study using the V1V2 DNA.

Nonneutralizing Fc-dependent Ab antiviral activities, including ADCC, ADCP, and C1q binding, the first step in activation of the classical complement cascade, were associated with reduced risk of infection in the RV144 human trial (2–6) as well as in the macaque SIV/SHIV infection model (2, 46, 75–80; reviewed in references 49 and 50). Our results show that priming with V1V2 DNA focuses the Ab responses preferentially on the V2 region, and these V2-specific Abs display potent effector functions, including ADCP and complement activation, features associated with a reduced risk of infection (16–23, 81). These data support the inclusion of V1V2 DNA priming vaccination to increase the magnitude and quality of the V2-specific Ab responses.

The use of a nucleic acid-based vaccine is a simple method allowing efficient expression of a structurally defined immunogen and results in the development of both humoral and cellular immunity (reviewed in references 34–36), which can be maintained for long periods and can be boosted by the same or heterologous boosting strategies (37). The use of DNA for priming immunizations provides a method to focus both Ab and cellular responses, in the absence of immune interference, on any desired protein region (30–33), resulting in the induction of immune responses with improved recognition of important epitopes associated with protection, as proposed for the V2 epitopes identified by the RV144 trial (2–6).

## MATERIALS AND METHODS

### Ethics statement.

All animals were cared for and procedures performed under a protocol approved by the Institutional Animal Care and Use Committee of Bioqual, Inc. (animal welfare assurance no. A3086-01; protocol number 15-008), and the United States Department of Agriculture (USDA certificate number 51-R0036). The 8 macaques (7 males and 1 female) in this study (F48) were managed according to the animal husbandry program, which aims at providing consistent and excellent care to nonhuman primates at the vivarium. This program operates based on the laws, regulations, and guidelines promulgated by the USDA (e.g., the Animal Welfare Act and its regulations and the Animal Care Policy Manual), Institute for Laboratory Animal Research (e.g., see reference 82), Public Health Service, National Research Council, Centers for Disease Control and Prevention, and the Association for Assessment and Accreditation of Laboratory Animal Care (AAALAC) International. The median age of the macaques was 5.3 years. The animals were negative for the Mamu-A*01, -B*08, and -B*17 alleles, and for simian T cell leukemia virus (STLV) (PCR negative and seronegative). Vaccinations were performed under anesthesia (ketamine administered at 10 mg/kg of body weight), and all efforts were made to minimize suffering. No adverse effects were found.

### Generation of DNA and protein vaccine components.

The HIV A/E.A244 V1V2 sequence (aa 140 to 219; GenBank accession number AWG42081), upon addition of the N-terminal tPA leader signal, was fused onto previously described scaffolds (25) (Protein Data Base [PDB] code 2F5K [plasmid 401H] and PDB code 2J9C [plasmid 417H]), and a FLAG tag was added at the C terminus to facilitate detection of *in vitro*-expressed proteins, resulting in plasmids 401H and 417H, respectively. The FLAG tag was removed from the V1V2 2J9C DNA (plasmid 418H) for the vaccine study. V1V2-TM (plasmid 447H) comprises the FLAG-tagged membrane-bound form of V1V2_A244_-2J9C generated upon fusion to the transmembrane domain from the platelet-derived growth factor receptor (PDGFR; aa 513 to 561; GenBank accession number AAA36427.1) used to generate stable cell lines for *in vitro* studies. Plasmid HIV AE.CM244 Env (GenBank accession number AAW57760) expresses the membrane-anchored gp145 (aa 1 to 720) that contains the transmembrane region (TM) but lacks the C-terminal cytoplasmic tail of gp41 (plasmid 416H). All plasmids contain RNA/codon-optimized genes inserted between the human cytomegalovirus (CMV) promoter and the BGH polyadenylation signal of the expression vector pCMV.kan (83). Endotoxin-free DNAs (catalog no. 12391; Qiagen, Valencia, CA) were prepared according to the manufacturer’s protocol. The CRF01_AE.CM244 gp120 Env protein (aa 1 to 512) expressed from plasmid 383H. gp120 was purified by lectin affinity column chromatography from supernatants collected from HEK293H cells (catalog no. 11631-017; Invitrogen) stably transfected with plasmid 383H and grown in serum-free medium in a hollow-fiber system (FiberCell Systems, Inc., Frederick, MD) as described previously (84).

### Expression analysis upon transient transfection in HEK293T cells.

DNA plasmids (250 ng) were transfected together with 6 μg Bluescript carrier DNA into HEK 293T cells by the CaCl_2_ coprecipitation method. Transfected cells and culture supernatants were harvested 48 h posttransfection for the analysis of protein expression. Cells were pelleted and resuspended in 1 ml of complete N1 lysis buffer (21). Equal proportions (1/200 fraction) of cell extracts or culture supernatants were loaded on denaturing 12% NuPAGE bis-Tris gels (catalog no. NP0342BOX; Thermo Fisher Scientific, MA) for V1V2 proteins or 10% NuPAGE bis-Tris gels (catalog no. NP0302PK2; Thermo Fisher Scientific) for gp145 and gp120 proteins. Proteins were transferred to nitrocellulose membranes and probed with horseradish peroxidase (HRP)-conjugated anti-FLAG antibody (1:5,000 dilution; catalog no. A8592; Sigma-Aldrich, St. Louis, MO) or with plasma from Env-vaccinated macaques followed by goat anti-monkey IgG/IgA/IgM (H+L)-HRP (1:10,000 dilution; catalog no. 43R-IG050hrp; Fitzgerald Industries International Inc., MA). The protein bands were visualized using an enhanced chemiluminescence (ECL) detection method (catalog no. RPN2232; GE HealthCare). To directly compare the expression levels of the proteins encoded by V1V2_A244_-2J9C and gp145_CM244_ DNAs, 250 ng of the plasmids were transfected in parallel plates seeded with HEK293T, and 24 h later, 1/200 of the cell-associated and extracellular fractions (total protein) were mixed and subjected to 2-fold serial dilution (1:2 to 1:16), and equal volumes were loaded on a denaturing 4 to 12% Bolt bis-Tris gel (catalog no. NW04122BOX; Thermo Fisher Scientific). Proteins were transferred to nitrocellulose membranes and probed with anti-V2 MAb CH58 (1:250 dilution; catalog no. 12550; NIH AIDS Reagent Program).

### Macaque vaccination schedule.

Two groups of Indian rhesus macaques (4 per group) were vaccinated following a regimen that included 2 DNA priming vaccinations (months 0 and 1) and 3 DNA-protein coimmunization booster vaccinations (months 2, 3, and 6). The DNA priming vaccine is composed of 2 mg DNA expressing either V1V2_A244_-2J9C (V1V2 DNA group) or CM244 gp145 (gp145 DNA group). The booster vaccination used for the V1V2-2J9C DNA-primed group is composed of 1 mg each of V1V2_A244_-2J9C DNA and CM244 gp145 DNA and 0.2 mg of gp120_A244_ protein. The gp145 DNA-primed group received 2 mg of CM244 gp145 DNA and 0.2 mg of gp120_A244_ protein. All DNA vaccines included 0.2 mg of rhesus macaque interleukin 12 (IL-12) DNA (plasmid AG157) as a molecular adjuvant (85). The DNA (1 ml) was delivered via the intramuscular route into the left (0.5 ml) and right (0.5 ml) inner thighs, followed by electroporation using the Elgen 1000 device (Inovio Pharmaceuticals Inc., Plymouth Meeting, PA). The booster DNA vaccines were coadministered with 0.2 mg CM244 gp120 protein, adjuvanted with 0.01 mg TLR-4 agonist (GLA-SE; Infectious Disease Research Institute, Seattle, WA) in 0.5 ml, which was delivered intramuscularly immediately following the DNA electroporation into the left (0.25 ml) and right (0.25 ml) inner thighs. Blood samples were analyzed at 2 weeks after the second priming vaccination and after the third booster vaccination.

### Immunoprecipitation.

Nondenaturing immunoprecipitation assays were performed under physiological salt conditions using supernatants from HEK293T cells transfected with 250 ng of V1V2_A244_-2J9C-FLAG DNA (plasmid 417H). Heat-inactivated plasma samples (5 μl) from immunized macaques were mixed with 20 μl of V1V2_A244_-2J9C-FLAG protein containing supernatant and 35 μl RBB400 buffer (15 mM HEPES, pH 7.9; 400 mM NaCl; 50 mM KCl; 0.1 mM EDTA; 0.2% Triton X-100; 10% glycerol) in a total reaction volume of 100 μl, and the mixtures were incubated overnight at 4°C. Next day, the immunocomplexes were incubated with 50 μl protein A-Sepharose beads (catalog no. 101141; Thermo Fisher Scientific) for 4 h. Bound proteins were collected by centrifugation at 13,000 rpm for 5 min, while the supernatants were collected to monitor the unbound protein fractions. The pellets were resuspended in 50 μl of 2× protein dye and incubated at 95°C for 5 min. Bound and unbound fractions were loaded onto 12% denaturing gels, analyzed by Western blotting, and visualized with anti-FLAG–HRP (1:5,000 dilution; Sigma-Aldrich).

### Surface staining of Env-expressing stable cell lines.

Stable HEK293H cell lines expressing membrane-anchored AE.CM244 V1V2-TM and gp145 from clade AE.CM244 and clade C.CH505.M5 (86) were generated. Cells were washed with PBS and harvested in PBS supplemented with 2 mM EDTA. One million cells were pelleted and mixed with 100 μl plasma from immunized macaques diluted with phosphate-buffered saline (PBS) or with 100 μl PBS containing 250 ng of various V2 Ab. For the gp145-expressing cell lines, 100 μl of 1:2-diluted plasma was used. For V1V2-expressing cell lines, 100 μl of 1:800 serially diluted immune plasma samples were used. The cells were incubated for 20 min at 4°C, washed with 10 ml PBS, and spun down. The pellets were resuspended in 100 μl of PBS containing 10 μl of phycoerythrin (PE)-conjugated anti-human IgG MAb known to cross-react with rhesus macaque IgG (clone G18-145; BD Biosciences, San Jose, CA). The cells were then washed in 10 ml PBS, pelleted, resuspended in 300 μl cold PBS, and placed on ice for analysis in a BD Fortessa cytometer. The following V2-specific MAbs were obtained from the NIH AIDS Reagent Program: PG9 (catalog no. 12149), PGT145 (catalog no. 12703), 697-30D (catalog no. 7371), CH58 (catalog no. 12550) and CH59 (catalog no. 12551). All the acquired data were analyzed using the FlowJo software platform (FlowJo, LLC, Ashland, OR).

### Ab measurements.

Vaccine-induced Abs were measured in serially diluted (4-fold starting at 1:50) heat-inactivated plasma samples by ELISA using 96-well plates coated with CM244 gp120 protein (50 ng/well) (Advanced Bioscience Lab, Rockville, MD). Samples were considered positive if the optical density (OD) was higher than the average of values obtained with naive macaque plasma +2 standard deviations. Endpoint titers were reported as the reciprocal of the highest dilution scoring positive. Abs to linear epitopes were also measured in plasma (1:50 dilution) using plates coated with 1 μg of peptides (20-mers overlapping by 14 aa) covering the V2 of CM244.

Plasma Abs to cyclic V2 (cV2) were measured by surface plasmon resonance (SPR; Biacore) using N-linked biotinylated cyclic HIV-1 V2 peptides (JPT Peptide Technologies, Berlin, Germany) captured on streptavidin-immobilized CM7 sensor chips as previously described (21, 87). Data analysis was performed using Biacore 4000 Evaluation software 4.1. The cV2 peptide sequences used are as follows: clade AE.92TH023, CSFNMTTELRDKKQKVHALFYKLDIVPIEDNTSSSEYRLINC; clade C.1086, CSFKATTELKDKKHKVHALFYKLDVVPLNGNSSSGEYRLINC.

A Luminex multiplex assay for binding of serum Abs was used as described elsewhere (29). HIV-1 antigens included recombinant gp120 from Immune Tech (New York, NY), and all peptides used bore an N-terminal 6× Lys-Gly (KG) linker. Peptides purchased from GenScript (Piscataway, NJ) included cV2_92TH023_ (CSFNMTTELRDKKQKVHALFYKLDIVPIEDNTSSSEYRLINC), cV2_1086_ (CSFKATTELKDKKHKVHALFYKLDVVPLNGSSSSGEYRLINC), and peptides spanning V2 (Fig. 5A). Antigens were covalently coupled to magnetic beads using a two-step carbodiimide reaction with the xMAP Ab coupling (AbC) kit according to the manufacturers’ instructions (Luminex, Austin, TX). Carboxylated xMAP beads were coupled to 4 μg protein/million beads (gp120s) or to 1 μg peptide/million beads. The coupled beads were counted, diluted to a concentration of 500,000 beads/ml, and stored at 4°C for up to 1 month prior to use. The concentrations used on the beads were established on the basis of the reactivity with MAbs, which were tested at concentrations ranging from 5 × 10^−6^ to 10 × 10^−6^ μg/ml (29). Beads coupled to bovine serum albumin (BSA) served as negative controls. A cocktail of MAbs composed of multiple V2i (697-30D, 830A, and 1393A), V2p (CH58), V3 (3869), and C5 (670, 1331A) MAbs (88–93) was used as a positive control. The conditions of the assay were established so that the readings used were from the midpoint (most sensitive part) of the sigmoidal titration curve, rather than binding using saturating conditions. This allowed interexperimental standardization. Each antigen was bound to a bead region with a different fluorescent profile. PE fluorescence was measured using a Luminex FlexMAP3D device with xPONENT 4.2 software. Nonhuman primate (NHP) serum was titrated at dilutions ranging from 1:100 to 150,000. Samples were tested in duplicate, and results are shown as mean fluorescence intensity (MFI).

The neutralizing Ab assay was conducted using Tat-regulated luciferase (Luc) reporter gene expression to quantify reductions in virus infection in TZM-bl cells. TZM-bl cells were obtained from the NIH AIDS Research and Reference Reagent Program, as contributed by John Kappes and Xiaoyun Wu (94–98). Assays were performed with HIV-1 Env-pseudotyped viruses (AE.TH023.6 and the mutant TH023.6N160K) produced in HEK293T cells essentially as previously described (99). Serum samples were heat inactivated at 56°C for 30 min, serially diluted in cell culture medium, and preincubated with pseudotyped virus (∼150,000 relative light unit equivalents) for 1 h at 37°C before addition of cells. Following a 48-h incubation, cells were lysed and Luc activity was determined using a microtiter plate luminometer and BriteLite Plus reagent (Perkin Elmer). Neutralization titers are the sample dilutions at which relative luminescence units (RLU) were reduced by 50% compared to RLU in virus control wells after subtraction of background RLU in cell control wells.

### ADCC.

ADCC was measured as described previously with the flow-based ADCC GranToxiLux assay (100, 101) using HIV subtype AE gp120_A244_Δ11_ coated CEM.NKR_CCR5_ as target cells (100). The results are reported as the Ab titer representing the dilution at which the titration curve intersected the 8% cutoff.

### ADCP.

ADCP was performed by measuring the uptake of HIV Env-coated fluorescent microspheres by the monocytic THP-1 cell line (ATCC; no. TIB-201), as described elsewhere (102). Briefly, 10 μl of biotinylated gp140_1086c_-coated bead suspension (1.8 × 10^6^ beads/ml) was mixed with 10 μl of 1:10 diluted heat-inactivated serum collected at prebleed and after the prime. After incubation at 37°C for 2 h, 2.5 × 10^4^ THP-1 cells were added to each well in a final volume of 200 μl and incubated for 1 h at 37°C. To prevent binding of the coated beads with CD4, the THP-1 cells were treated with 1 μg of gp140_1086c_ for 1 h at 37°C before addition to the bead-serum mixture. After a 1-h incubation at 37°C, the cells were transferred to tubes and acquired by flow cytometry. The phagocytosis score was calculated for both preimmunized and immunized sera by multiplying the percentage of bead-positive cells by mean fluorescent intensity (MFI) and dividing by 10^6^. Fold differences between values before and after priming were calculated by dividing the scores obtained after the prime and the prebleed.

### C1q binding ELISA to measure complement activation.

C1q binding to vaccine-induced V2-specific Abs was measured by ELISA using a modified protocol described previously (81, 103). Immulon 4HBX 96-well plates (no. 3855; Thermo Scientific) were coated with 2 μg/ml of cyclic V2_1086_ peptide (CSFKATTELKDKKHKVHALFYKLDVVPLNGSSSSGEYRLINC). Antigens were coated with 100 μl per well in 50 mM sodium carbonate-bicarbonate buffer, overnight at 4°C. Plates were washed three times with 150 μl wash buffer 1 per well (1× PBS plus 0.05% BSA), followed by a blocking step with 150 μl per well of 1× PBS plus 7.5% BSA for 1.5 h at room temperature. Plates were washed three times with 150 μl per well with wash buffer 1. Plasma samples were heat inactivated at 56°C for 30 min and diluted 2-fold at dilutions ranging from 50× to 400×. A 130-μl portion of the serially diluted samples was transferred to the assay plate and incubated for 1.5 h at room temperature. Plates were washed three times with 150 μl of wash buffer 1 and incubated with 10 μg/ml of C1q (no. C1740; Sigma) at 100 μl per well for 1.5 h at room temperature with shaking at 500 rpm. The C1q was diluted in 1× PBS plus 0.5% BSA and 0.05% Tween 20. After washing four times with 200 μl per well with wash buffer 2 (1× PBS plus 0.05% Tween 20), bound antibodies were detected with mouse monoclonal IgG1 anti-human C1q-HRP (no. sc-53544; Santa Cruz) with 100 μl per well at 10 μg/ml, diluted in 1× PBS plus 0.5% BSA and 0.05% Tween 20. Anti-C1–HRP was incubated for 1 h at room temperature with shaking at 500 rpm. After washing with 200 μl per well with wash buffer 2, 100 μl per well of 1-Step Ultra TMB (3,3′,5,5′-tetramethylbenzidine) substrate (no. 34028; Thermo Scientific) was added and incubated for 15 min in the dark. The reaction was stopped with the addition of 100 μl per well of 1 N hydrochloric acid. The plates were read at 450 nm in a BioTek PowerWave HT plate reader. Assays were standardized with positive-control (CAP228 16H) and negative-control (3685) MAbs included with each assay. Background from prebleeds was subtracted from the value obtained 2 weeks after the prime and after the last boost, and the area under the curve (AUC) was determined.

### Statistical analyses.

All data were analyzed using GraphPad Prism version 8.0 for MacOS X (GraphPad Software, Inc., La Jolla, CA) with the exception of the C1q data, which were analyzed using GraphPad Prism 7.03 software.
